# The Role of Matrix Metalloproteinases in Orthodontics, Dental Trauma, Restorative Dentistry, and Endodontics: Molecular Mechanisms and Clinical Implications

**DOI:** 10.3390/ijms27114800

**Published:** 2026-05-26

**Authors:** Renata Ławicka, Kinga Królikowska, Katarzyna Błaszczak, Zuzanna Borawska, Monika Zbucka-Krętowska, Sławomir Ławicki, Magdalena Nowosielska

**Affiliations:** 1 The “Karedent” Dental Clinic, Bukowskiego 1/U3, 15-066 Białystok, Poland; 2 Department of Population Medicine and Lifestyle Diseases Prevention, Medical University of Białystok, Waszyngtona 13a, 15-269 Białystok, Poland; 3 University Hospitals Coventry and Warwickshire Trust, Clifford Bridge Road, Walsgrave, Coventry CV2 2DX, UK; 4 Department of Gynaecological Endocrinology and Adolescent Gynaecology, Medical University of Białystok, M. Skłodowskiej-Curie 24a, 15-276 Białystok, Poland; 5 Department of Gerostomatology, Medical University of Białystok, Akademicka 3, 15-267 Białystok, Poland

**Keywords:** matrix metalloproteinases (MMPs), tissue inhibitors of metalloproteinases (TIMPs), orthodontic tooth movement, root resorption, restorative dentistry, adhesive systems, hybrid layer, pulp inflammation, endodontics, biomarkers, tissue regeneration

## Abstract

Matrix metalloproteinases (MMPs) are zinc-dependent proteolytic enzymes involved in extracellular matrix remodelling in oral and dental tissues, including the periodontal ligament, alveolar bone, dentin, dental pulp, and periapical tissues. This narrative review summarises selected evidence on the role of MMPs and tissue inhibitors of metalloproteinases (TIMPs) in orthodontic tooth movement, dental trauma and root resorption, restorative adhesive dentistry, and pulp/periapical disease. Particular attention is given to signalling pathways that regulate MMP/TIMP activity, including nuclear factor kappa B (NF-κB), mitogen-activated protein kinase (MAPK), Wnt/β-catenin, and transforming growth factor beta (TGF-β)/Smad-related mechanisms. The review also discusses the biomarker potential and translational status of MMP-targeted strategies. Across clinical contexts, MMP activity contributes to both matrix degradation and tissue repair, and its biological effect depends on local stimuli, TIMP-mediated regulation, pathway crosstalk, and the stage of disease or treatment.

## 1. Introduction

Matrix metalloproteinases (MMPs) are a family of zinc-dependent endopeptidases that participate in the remodelling and degradation of extracellular matrix (ECM) components in oral and dental tissues. In dentistry, their relevance extends across the periodontal ligament, alveolar bone, dentin, dental pulp, and periapical tissues, where they contribute to physiological tissue turnover as well as inflammation-associated matrix breakdown [1]. Based on substrate preference and structural characteristics, MMPs are commonly classified into collagenases, gelatinases, stromelysins, matrilysins, membrane-type MMPs (MT-MMPs), and other MMPs [2]. The major groups and representative substrates are summarised in Table 1. The table should be interpreted as a representative overview rather than as a complete enzyme–substrate map, because substrate specificity differs between individual MMPs, activation states, and tissue microenvironments.

MMP activity is regulated by transcriptional control, proteolytic activation of proenzymes, compartmentalisation within the ECM, and inhibition by tissue inhibitors of metalloproteinases (TIMPs). TIMP isoforms are not interchangeable inhibitors; TIMP-1 to TIMP-4 differ in tissue distribution, binding characteristics, extracellular localisation, and MMP-independent functions [3,4].

The value of this review is not the proposal of nuclear factor kappa B (NF-κB), mitogen-activated protein kinase (MAPK), Wnt/β-catenin, or transforming growth factor beta (TGF-β)/Smad pathways as novel mechanisms. These pathways have been described previously in matrix biology and oral/dental research. Rather, this narrative review compares how MMP/TIMP-related ECM remodelling appears across several clinical contexts in dentistry and how similar molecular mediators may produce different biological outcomes depending on tissue type, stimulus, and disease stage [5].

The purpose of this narrative review is to summarise selected evidence on how MMPs and TIMPs contribute to ECM remodelling across four clinically relevant areas of dentistry: orthodontic tooth movement, dental trauma and root resorption, restorative adhesive dentistry, and pulpal/periapical disease. The review also aims to compare shared and context-specific molecular mechanisms, including inflammatory, mechanotransductive, and regenerative signalling pathways, and to discuss the biomarker and therapeutic relevance of MMP modulation in these settings.

### Literature Search Strategy

This narrative review was based on a literature search performed in PubMed/MEDLINE, Scopus, and Web of Science. The search included combinations of the following terms: “matrix metalloproteinases”, “MMP”, “TIMPs”, “orthodontic tooth movement”, “dental trauma”, “root resorption”, “adhesive dentistry”, “hybrid layer”, “dentin bonding”, “pulpitis”, “periapical lesions”, “endodontics”, “regenerative endodontics”, “TGF-β/Smad”, “NF-κB”, “MAPK”, “Wnt/β-catenin”, “chlorhexidine”, “doxycycline”, “host modulation therapy”, “silver diamine fluoride”, and “collagen cross-linkers”. The search focused primarily on English-language articles, with emphasis on publications from the last 10 years, while older landmark studies were retained when they provided foundational mechanistic or clinical information. Original studies, systematic reviews, narrative reviews, and clinically relevant translational studies were considered. Articles were selected based on relevance to MMP/TIMP biology, ECM remodelling, biomarker potential, and therapeutic modulation of MMP activity in oral and dental tissues. Because this was a narrative review, no formal systematic screening or risk-of-bias assessment was performed; articles unrelated to MMP/TIMP biology in oral and dental tissues, non-peer-reviewed sources, conference abstracts, and non-English papers without an English-language abstract were excluded.

## 2. Molecular Regulation of MMP/TIMP Activity in Oral and Dental Tissues

**Figure 1:**
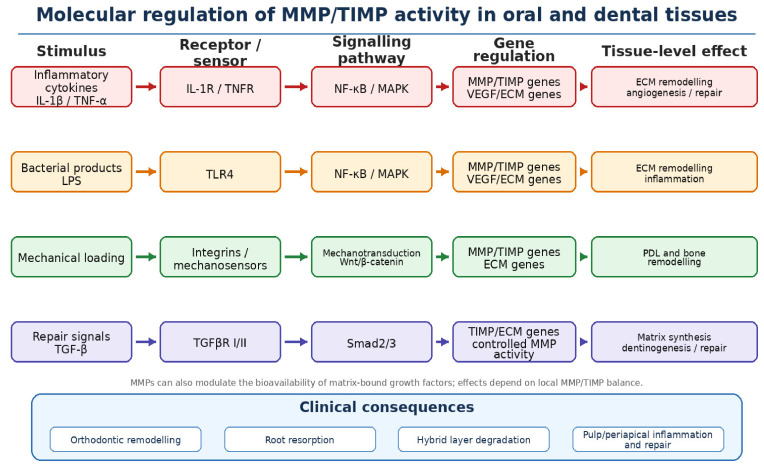
Molecular regulation of MMP/TIMP activity in oral and dental tissues. The revised schematic shows a pathway-based sequence from extracellular stimuli and receptors/sensors to intracellular signalling, gene regulation, tissue-level effects, and clinical consequences. Abbreviations: ECM, extracellular matrix; IL-1β, interleukin-1 beta; LPS, lipopolysaccharide; MAPK, mitogen-activated protein kinase; MMP, matrix metalloproteinase; NF-κB, nuclear factor kappa B; TGF-β, transforming growth factor beta; TIMP, tissue inhibitor of metalloproteinases; TNF-α, tumour necrosis factor alpha; VEGF, vascular endothelial growth factor.

MMP/TIMP activity in oral and dental tissues is regulated by inflammatory mediators, mechanical loading, microbial products, and repair-associated signals. These stimuli activate signalling pathways that converge on gene expression, proenzyme activation, ECM degradation, ECM synthesis, angiogenesis, and tissue repair (Figure 1).

### 2.1. NF-κB, MAPK, and Microbial/Inflammatory Stimuli

NF-κB-related signalling is activated by inflammatory cytokines and is associated with increased expression of several MMPs. In dental cells, interleukin-1α has been shown to regulate MMP-9, while tumour necrosis factor alpha (TNF-α)-related responses may involve both NF-κB and MAPK signalling depending on cell type and stimulus [6]. JNK1/2 signalling contributes to TNF-α-enhanced MMP-3 production in human dental pulp fibroblast-like cells, whereas p38 MAPK and NF-κB signalling have been implicated in TNF-α-induced MMP-3 production in cementoblast-like cells [7,8].

Microbial products should be discussed cautiously. Lipopolysaccharide (LPS) derived from Gram-negative bacteria is widely used to model inflammatory responses in dental pulp cells; however, extrapolation from periodontal biofilm-driven inflammation or in vitro pulpal models to clinical endodontic infections requires caution [9].

### 2.2. Wnt/β-Catenin and Mechanotransduction

Mechanical loading in orthodontic tooth movement activates mechanotransductive responses in periodontal ligament and alveolar bone. The glycogen synthase kinase-3 beta (GSK-3β)/β-catenin axis has been linked to osteoclast differentiation, receptor activator of nuclear factor kappa-B ligand (RANKL)-related signalling, and MMP-9 expression during compressive force-induced alveolar bone resorption [10].

### 2.3. TGF-β/Smad Signalling and Pathway Crosstalk

TGF-β/Smad signalling is relevant to ECM remodelling in oral and dental tissues and was added to address the molecular pathway gap identified during review. TGF-β signalling influences fibroblast and odontoblast-related responses, ECM production, TIMP expression, angiogenesis, and reparative dentinogenesis. In human odontoblasts and dental pulp cells, TGF-β1 down-regulates MMP-8 expression, suggesting that TGF-β signalling can modulate proteolytic activity as well as matrix synthesis [11].

Dental pulp, odontoblasts, and dentin contain dynamic TGF-β signalling networks, and latent TGF-β can be activated by MMP-related mechanisms in the dentin–pulp complex [12]. TGF-β1 has also been shown to increase collagen content, procollagen I, and TIMP-1 production in human dental pulp cells through MEK/ERK and ALK5/Smad-related signalling [13]. Experimental root formation studies further support TGF-β1/Smad2/3 as a pathway involved in odontoblast differentiation and tooth root formation [14].

Crosstalk between TGF-β/Smad and inflammatory pathways such as NF-κB and MAPK may help determine whether MMP activity contributes predominantly to tissue destruction, adaptive remodelling, or repair. In inflamed dental tissues, NF-κB/MAPK-related signalling may favour inflammatory mediator and MMP expression, whereas TGF-β/Smad signalling is more closely associated with ECM synthesis, TIMP production, and reparative responses.

### 2.4. TIMP Isoforms and Local MMP/TIMP Balance

TIMP isoforms should be interpreted individually. TIMP-1 and TIMP-2 are frequently discussed in oral inflammatory conditions; TIMP-2 also participates in MT1-MMP-dependent proMMP-2 activation. TIMP-3 is more tightly associated with the ECM and has a broader metalloproteinase inhibitory profile, while TIMP-4 is less extensively studied in dental tissues. Experimental apical periodontitis models show that MMP-1, MMP-2, MMP-8, MMP-9, TIMP-1, and TIMP-2 vary over lesion development, emphasising the importance of temporal and tissue-specific MMP/TIMP ratios [15]. The main TIMP isoforms and their relevance to MMP-dependent ECM remodelling are summarised in Table 2.

## 3. Metalloproteinases in Orthodontic Tooth Movement

**Figure 2:**
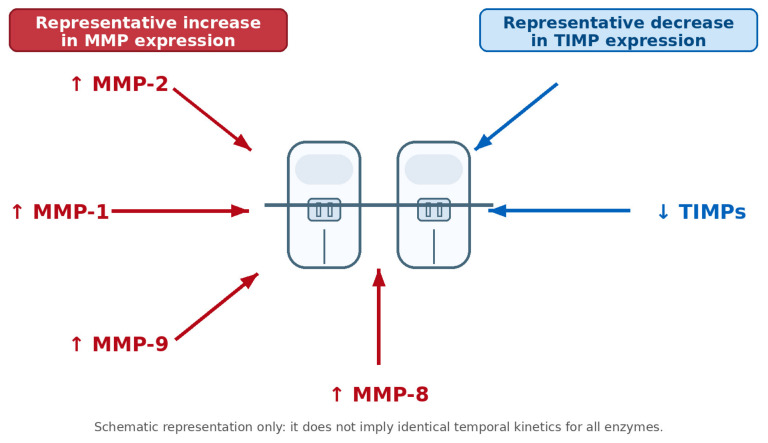
Selected MMP/TIMP changes associated with orthodontic tooth movement. The schematic illustrates representative involvement of MMP-1, MMP-2, MMP-8, MMP-9 and TIMPs in periodontal remodelling. It does not imply identical temporal kinetics for all enzymes. Abbreviations: MMP, matrix metalloproteinase; TIMP, tissue inhibitor of metalloproteinases.

Orthodontic tooth movement requires coordinated remodelling of the periodontal ligament and alveolar bone. Mechanical loading generates compression and tension zones, leading to cytokine release, osteoclast/osteoblast activity, and ECM turnover in which MMPs and TIMPs participate [16,17]. The representative MMP/TIMP changes associated with orthodontic tooth movement are shown in Figure 2.

Collagenases such as MMP-1 and MMP-8 contribute to the degradation of fibrillar collagen within the periodontal ligament, while gelatinases such as MMP-2 and MMP-9 degrade denatured collagen and basement membrane-related substrates. Grant et al. reported early changes in cytokines, MMP-9, TIMPs, RANKL, and osteoprotegerin (OPG) during orthodontic tooth movement, but the kinetics of individual enzymes depend on sampling time and experimental protocol [17].

Increased MMP-8 levels in peri-miniscrew crevicular fluid have been observed shortly after mini-screw placement [18]. These findings may indicate an early local tissue response to mini-screw insertion; however, the available data do not allow a definitive conclusion regarding the exact biological mechanism or the extent of subsequent tissue adaptation.

Experimental inhibition of MMP activity has been shown to reduce orthodontic tooth movement in animal models, including local delivery of broad-spectrum MMP inhibitors and systemic administration of chemically modified tetracycline-3 (CMT-3) [19,20]. These data support mechanistic involvement of MMPs in orthodontic remodelling, although direct clinical application of MMP inhibition in orthodontics remains experimental.

## 4. Metalloproteinases in Dental Trauma and Root Resorption

**Figure 3:**
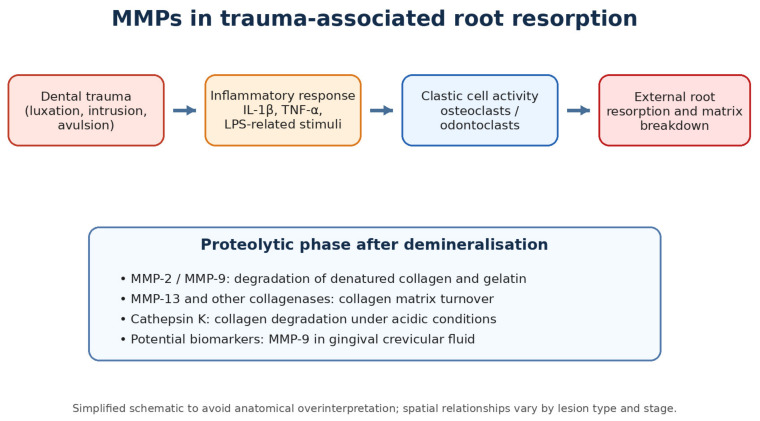
Simplified pathway of MMP involvement in trauma-associated root resorption. The figure was redesigned as a mechanistic flowchart to avoid anatomical overinterpretation. Abbreviations: IL-1β, interleukin-1 beta; LPS, lipopolysaccharide; MMP, matrix metalloproteinase; TNF-α, tumour necrosis factor alpha.

Dental trauma, including intrusion, extrusion, avulsion, and luxation injuries, damages periodontal and pulpal tissues and may initiate inflammatory root resorption. Root resorption is generally mediated by clastic cells and is influenced by injury type, infection, inflammatory mediators, and the integrity of protective cementoblastic and periodontal ligament barriers [21,22,23]. The simplified pathway of MMP involvement in trauma-associated root resorption is shown in Figure 3.

This biomarker perspective should be integrated with established dental traumatology guidance rather than interpreted as an independent treatment pathway. The 2020 International Association of Dental Traumatology (IADT) guidelines emphasise rapid diagnosis, injury-specific emergency management, appropriate splinting or replantation when indicated, endodontic decision-making according to injury type and root development, and scheduled clinical and radiographic follow-up to detect complications such as inflammatory root resorption [24,25]. In this context, MMP-9 and related biomarkers are best viewed as potential adjunctive tools for future risk stratification or monitoring, not as replacements for guideline-based clinical management. Following demineralisation of mineralised tissues, organic matrix degradation involves enzymes such as MMPs and cathepsin K. MMP-2 and MMP-9 have been reported in replanted teeth with external root resorption, while gingival crevicular fluid studies after traumatic dental injury have evaluated inflammatory mediators, including MMP-9, as potential adjunctive biomarkers of trauma-associated external inflammatory root resorption [26,27].

The proteolytic phase of resorption should be understood in relation to osteoclast/odontoclast activity. Acidification promotes mineral dissolution, while proteases including cathepsin K and MMPs degrade the exposed organic matrix [28,29].

At present, MMP-targeted management of post-traumatic root resorption remains mainly conceptual or translational. Clinically, early diagnosis, infection control, appropriate splinting, and follow-up imaging remain central, while MMP-9 and related markers may support future risk stratification rather than replace established clinical assessment.

## 5. Metalloproteinases in Restorative and Adhesive Dentistry

### 5.1. Hybrid Layer Degradation and MMP Activation

The durability of adhesive restorations depends on the formation and long-term stability of the hybrid layer, which results from resin monomer infiltration into demineralised dentin [30,31]. Incomplete infiltration leaves collagen fibrils susceptible to hydrolysis and enzymatic degradation, and endogenous dentinal MMPs, especially MMP-2 and MMP-9, contribute to degradation of exposed collagen within the adhesive interface [32,33].

**Figure 4:**
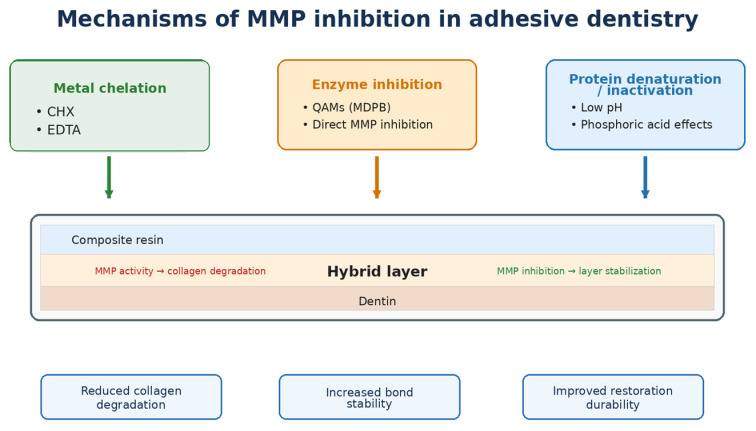
Mechanisms of MMP inhibition in adhesive dentistry. MMP activity within the hybrid layer may be reduced by metal ion chelation, direct enzymatic inhibition, and protein denaturation/inactivation under selected conditions. These mechanisms may reduce collagen degradation, improve hybrid layer stability, and increase restoration durability. Abbreviations: CHX, chlorhexidine; EDTA, ethylenediaminetetraacetic acid; MDPB, 12-methacryloyloxydodecylpyridinium bromide; MMP, matrix metalloproteinase; QAMs, quaternary ammonium compounds.

The relationship between pH and dentinal MMP activity is dynamic. Self-etching systems may increase gelatinolytic/collagenolytic activity, while phosphoric acid etching can transiently affect protease activity; subsequent adhesive procedures may reactivate previously inactivated endogenous proteolytic activity. Thus, hybrid layer degradation should be interpreted as a process influenced by etching strategy, adhesive composition, water sorption, and long-term enzymatic reactivation, rather than by low pH alone [34,35,36]. The main mechanisms of MMP inhibition within the adhesive interface are summarised in Figure 4.

### 5.2. MMP-Modulating Strategies and Adhesive Systems

Chlorhexidine (CHX) has been widely studied as an MMP inhibitor in adhesive dentistry. Original experimental work demonstrated that CHX inhibits MMP-2, MMP-8, and MMP-9 activity, while dental studies and reviews have assessed its use for reducing hybrid layer degradation [37,38,39]. However, the clinical durability of CHX pretreatment is limited by gradual leaching and loss of effect over time.

Quaternary ammonium compounds (QAMs), including 12-methacryloyloxydodecylpyridinium bromide (MDPB), have been investigated as polymerisable antimicrobial and MMP-modulating agents. QAM-containing adhesive systems may provide longer-lasting inhibition because the active moiety can be incorporated into the adhesive matrix, although concerns regarding formulation, cytotoxicity, and long-term biological performance remain under investigation [40,41,42].

Additional approaches include chelating agents, biomimetic remineralisation, and collagen stabilisation. Biomimetic approaches aim to protect exposed collagen and facilitate mineral deposition within the hybrid layer [43]. More recent evidence has evaluated natural and synthetic collagen cross-linkers, including proanthocyanidins, epigallocatechin-3-gallate (EGCG), riboflavin-based systems, and carbodiimide, as strategies to enhance resin–dentin bond durability by stabilising collagen and reducing susceptibility to proteolytic degradation [44,45].

Silver diamine fluoride (SDF) has also been reported to inhibit dentinal MMP activity in vitro in a concentration-dependent manner, but its role in adhesive dentistry should be interpreted within the broader context of caries control, dentin preservation, discoloration, and effects on bonding protocols [46].

Adhesive systems have progressed from multi-step etch-and-rinse systems to simplified self-etch systems and contemporary universal adhesives. Universal adhesives allow etch-and-rinse, self-etch, or selective enamel etching strategies and frequently contain functional monomers such as 10-methacryloyloxydecyl dihydrogen phosphate (10-MDP). Their clinical performance depends not only on immediate bond strength but also on hydrolytic stability, substrate condition, aging protocol, and enzymatic degradation within the hybrid layer [47,48].

The evolution and main characteristics of dental adhesive systems are summarised in Table 3.

## 6. Metalloproteinases in Pulpal and Periapical Disease/Endodontics

MMPs in the dentin–pulp complex and periapical tissues have dual roles. They can contribute to ECM degradation during pulpitis and apical periodontitis, but also participate in angiogenesis, matrix release of bioactive molecules, and reparative dentinogenesis [49,50].

MMP-8 and related inflammatory mediators have been measured in reversible and irreversible pulpitis, and MMP-8 changes have also been detected in pulp tissue and gingival crevicular fluid from symptomatic irreversible pulpitis teeth [51,52]. MMP-3 may accelerate wound healing after pulp injury and is involved in matrix remodelling, angiogenesis, and reparative processes [53].

Active MMP-9 in pulpal blood has been associated with pulpotomy outcome in permanent mature teeth with irreversible pulpitis, suggesting prognostic biomarker potential, although larger prospective studies are needed before routine clinical use can be recommended [54]. In apical periodontitis, MMP/TIMP dynamics, including MMP-1, MMP-2, MMP-8, MMP-9, TIMP-1, and TIMP-2, have been implicated in the periapical immune response and lesion development [15].

MMP-8 has been detected in pulpal and periapical inflammation and periapical root-canal exudate, supporting the broader concept that MMPs may reflect local inflammatory activity in endodontic disease [55]. Genetic polymorphism studies have also linked MMPs and TIMPs with periapical lesion biology, but the clinical significance of these associations remains incompletely defined [49].

## 7. Cross-Context Clinical Synthesis and Future Directions

Across orthodontics, trauma, restorative dentistry, and endodontics, MMPs should not be interpreted simply as destructive enzymes. Their clinical impact depends on context: controlled remodelling is required for orthodontic tooth movement, excessive matrix degradation contributes to root resorption and hybrid layer failure, and balanced proteolysis can participate in pulp repair and dentinogenesis.

A cross-context comparison of MMP/TIMP involvement, biomarker potential, and clinical translation status across the discussed dental conditions is presented in Table 4.

Although periodontitis is not the primary focus of this review, it provides the strongest clinical evidence for oral-fluid MMP biomarkers, particularly MMP-8 in gingival crevicular fluid, mouthrinse, and saliva [56,57]. This review therefore uses periodontal data as a translational reference framework while focusing on orthodontics, trauma-related resorption, restorative dentistry, and endodontics.

From a translational perspective, MMP-targeted approaches differ substantially in clinical validation. Sub-antimicrobial-dose doxycycline is the most clinically established host-modulation strategy targeting MMP activity, particularly as an adjunct to scaling and root planing in periodontal therapy [58,59]. In contrast, most MMP-modulating approaches in restorative dentistry, trauma-related resorption, and regenerative endodontics remain experimental, in vitro, or early translational strategies.

Future research should define condition-specific MMP/TIMP signatures, clarify pathway crosstalk among NF-κB, MAPK, Wnt/β-catenin, and TGF-β/Smad signalling, validate non-invasive biomarkers such as MMP-8 and MMP-9, and develop locally targeted strategies that limit pathological matrix degradation while preserving physiological remodelling and repair.

## 8. Conclusions

The main clinical message of this review is that MMPs and TIMPs should be interpreted as context-dependent regulators of tissue remodelling rather than as universally harmful enzymes or simple therapeutic targets. In orthodontic tooth movement, MMP activity mainly reflects controlled periodontal ligament and alveolar bone remodelling; in traumatic dental injury, MMP-9 and related biomarkers may help future risk stratification for root resorption but should not replace guideline-based diagnosis, treatment, and follow-up.

In restorative adhesive dentistry, protection of exposed dentinal collagen and reduction of enzymatic hybrid layer degradation remain practical strategies for improving restoration durability. In pulpal and periapical disease, MMP/TIMP patterns may provide information on inflammation, prognosis, and repair potential, but their routine diagnostic or therapeutic use requires further clinical validation.

Therefore, the most clinically relevant approach is not complete MMP suppression, but context-specific modulation that limits pathological matrix breakdown while preserving physiological remodelling and reparative processes. At present, clinically established MMP-targeted therapy is strongest in periodontal host modulation, whereas applications in orthodontics, dental trauma, adhesive dentistry, and endodontics remain mainly experimental or translational.

## Figures and Tables

**Table 1:** Classification of matrix metalloproteinases (MMPs) and representative substrates. The listed substrates are representative examples for each MMP group and should not be interpreted as substrates of every individual enzyme within the group [2].

MMP Group	Main Members	Representative Substrates/Functions (Not Exhaustive)
Collagenases	MMP-1, MMP-8, MMP-13	Primarily fibrillar collagens, especially types I, II and III; additional ECM substrates vary by enzyme and tissue context.
Gelatinases	MMP-2, MMP-9	Gelatin, denatured collagens, basement membrane components such as collagen type IV and laminin, elastin and several non-collagenous ECM proteins.
Stromelysins	MMP-3, MMP-10, MMP-11	Proteoglycans, laminin, fibronectin, non-fibrillar collagens and activation of selected proMMPs, particularly by MMP-3.
Matrilysins	MMP-7, MMP-26	Broad substrate spectrum including gelatin, casein, elastin, fibronectin and selected proMMPs; activity is context- and isoform-dependent.
Membrane-type MMPs (MT-MMPs)	MMP-14, MMP-15, MMP-16, MMP-17, MMP-24, MMP-25	Pericellular ECM turnover, collagen/gelatin/fibronectin degradation depending on isoform, and activation of proMMP-2 and other proMMPs.
Other MMPs	MMP-12, MMP-19, MMP-20, MMP-21, MMP-23, MMP-27, MMP-28	Tissue-specific substrate profiles; examples include elastin (MMP-12), amelogenin (MMP-20), casein, laminin, nidogen and tenascin.

Abbreviations: ECM, extracellular matrix; MMP, matrix metalloproteinase; MT-MMP, membrane-type matrix metalloproteinase; TIMP, tissue inhibitor of metalloproteinases.

**Table 2:** Main tissue inhibitors of metalloproteinases (TIMPs) and their relevance to ECM remodelling [3,4,15].

TIMP Isoform	Main Biochemical Features	Selected MMP-Related Actions	Relevance in Oral and Dental Tissues
TIMP-1	Soluble glycoprotein inhibitor of several soluble MMPs; often discussed in inflammatory remodelling.	Inhibits several collagenases and gelatinases, including MMP-9-related activity.	Contributes to the local MMP/TIMP balance in inflamed oral tissues; potential biomarker relevance in inflammatory conditions.
TIMP-2	MMP inhibitor that also participates in MT1-MMP-dependent proMMP-2 activation.	Regulates MMP-2 activation as well as inhibition, depending on local concentration and MT1-MMP context.	Important for gelatinase regulation and pericellular ECM turnover in remodelling tissues.
TIMP-3	ECM-associated inhibitor with broader metalloproteinase inhibitory profile.	Locally restricts matrix degradation and inflammatory proteolysis through strong matrix binding.	Potentially relevant to tissue homeostasis where local ECM-bound inhibition is required; dental-specific evidence remains limited.
TIMP-4	More restricted tissue distribution and less extensively studied in dental tissues.	Can inhibit selected MMPs, but its oral/dental roles are less well characterised.	Potential regulatory role in tissue remodelling; requires further dental-specific investigation.

Abbreviations: ECM, extracellular matrix; MMP, matrix metalloproteinase; MT1-MMP, membrane-type 1 matrix metalloproteinase; TIMP, tissue inhibitor of metalloproteinases.

**Table 3:** Evolution and classification of dental adhesive systems. The table was prepared by the authors based on current adhesive dentistry literature [31,32,33,47,48]. Bond strength values are representative approximate ranges synthesised from the cited literature and may vary according to substrate, testing method, aging protocol, and adhesive formulation; they should not be interpreted as standardised values directly comparable across studies.

Generation/Category	Etching Strategy	Number of Steps	Main Components	Key Characteristics	Representative Bond Strength (MPa)
IV (total-etch, 3-step)	Etch-and-rinse (ER)	3	Etchant + primer + adhesive	Reference multi-step approach with high durability; technique-sensitive and moisture-dependent.	20–25
V (total-etch, 2-step)	Etch-and-rinse (ER)	2	Etchant + combined primer/adhesive	Simplified ER protocol; good immediate bond strength but increased sensitivity to moisture control.	20–25
VI (self-etch, 2-step)	Self-etch (SE)	2	Self-etch primer + adhesive	Less aggressive dentin demineralisation and reduced technique sensitivity compared with ER systems.	17–22
VII (self-etch, 1-step)	Self-etch (SE)	1	All-in-one adhesive	Simplified application; may be more prone to phase separation, water sorption and reduced long-term durability.	17–22
VIII/universal adhesives	ER/SE/selective enamel etch	1 or 2	Single-bottle or dual-component systems with functional monomers	Multi-mode use; often contains 10-MDP or other functional monomers to enhance chemical interaction with hydroxyapatite.	25–35
Modern universal adhesives	ER/SE/selective enamel etch	1	Multi-mode adhesive with functional monomers ± silane	Versatile use for direct and indirect restorative procedures; performance depends on substrate pretreatment and ageing conditions.	25–40

Abbreviations: ER, etch-and-rinse; SE, self-etch; 10-MDP, 10-methacryloyloxydecyl dihydrogen phosphate.

**Table 4:** Cross-context comparison of MMP/TIMP involvement in dental clinical conditions.

Clinical Context	Main MMPs/TIMPs	Dominant Stimuli/Pathways	Main Biological Effect	Biomarker Potential	Clinical Translation Status
Orthodontic tooth movement	MMP-1, MMP-2, MMP-8, MMP-9, TIMPs	Mechanical loading; RANKL/OPG; Wnt/β-catenin; MAPK-related signalling.	Periodontal ligament and alveolar bone remodelling.	MMP-8 and MMP-9 in gingival crevicular fluid or saliva may reflect remodelling activity.	Primarily observational and experimental; MMP inhibition can reduce tooth movement in animal models.
Dental trauma and root resorption	MMP-2, MMP-9, MMP-13, cathepsin K, TIMPs	Trauma-induced inflammation; clastic cell activity; cytokine signalling.	Organic matrix degradation after mineral dissolution and progression of external root resorption.	MMP-9 may indicate trauma-associated resorptive activity.	Biomarker potential; targeted MMP modulation remains clinically limited.
Restorative/adhesive dentistry	MMP-2, MMP-8, MMP-9; CHX-, QAM-, SDF- and cross-linker-sensitive collagenolytic activity	Acid etching; exposed collagen; water sorption; hybrid layer ageing.	Hybrid layer degradation and deterioration of resin–dentin bond durability.	Limited diagnostic use; mainly mechanistic and material-performance relevance.	CHX, QAMs, cross-linkers, SDF and biomimetic remineralisation are mostly in vitro, ex vivo or translational strategies.
Pulpal and periapical disease/endodontics	MMP-1, MMP-2, MMP-3, MMP-8, MMP-9, TIMP-1, TIMP-2	Cytokines; LPS-related inflammatory models; NF-κB/MAPK; TGF-β/Smad-related repair signalling.	Matrix degradation, angiogenesis, reparative dentinogenesis and periapical bone resorption.	MMP-8 and MMP-9 may support diagnostic/prognostic assessment.	Emerging biomarker and regenerative relevance; routine clinical protocols are not established.
Periodontal literature as translational reference framework	MMP-8, MMP-9, TIMPs	Biofilm-driven inflammation; host response; cytokine-mediated proteolysis.	Periodontal collagen degradation and tissue destruction.	Strongest oral-fluid biomarker evidence, especially active MMP-8.	Sub-antimicrobial-dose doxycycline is the most clinically validated MMP-targeted host-modulation approach.

## Data Availability

No new data were created or analysed in this study. Data sharing is not applicable to this article.

## Abbreviations

The following abbreviations are used in this manuscript:

Abbreviation	Definition
10-MDP	10-methacryloyloxydecyl dihydrogen phosphate
CHX	chlorhexidine
ECM	extracellular matrix
EDTA	ethylenediaminetetraacetic acid
ER	etch-and-rinse
GCF	gingival crevicular fluid
GSK-3β	glycogen synthase kinase-3 beta
IL-1β	interleukin-1 beta
LPS	lipopolysaccharide
MAPK	mitogen-activated protein kinase
MDPB	12-methacryloyloxydodecylpyridinium bromide
MMP	matrix metalloproteinase
MT-MMP	membrane-type matrix metalloproteinase
NF-κB	nuclear factor kappa B
PDL	periodontal ligament
QAM	quaternary ammonium compound
RANKL	receptor activator of nuclear factor kappa-B ligand
SDD	sub-antimicrobial-dose doxycycline
SDF	silver diamine fluoride
SE	self-etch
TGF-β	transforming growth factor beta
TIMP	tissue inhibitor of metalloproteinases
TNF-α	tumour necrosis factor alpha
VEGF	vascular endothelial growth factor
